# Mobile-Based Nutrition Counseling and Unconditional Cash Transfers for Improving Maternal and Child Nutrition in Bangladesh: Pilot Study

**DOI:** 10.2196/mhealth.8832

**Published:** 2018-07-18

**Authors:** Tanvir M Huda, Ashraful Alam, Tazeen Tahsina, Mohammad Mehedi Hasan, Jasmin Khan, Mohammad Masudur Rahman, Abu Bakkar Siddique, Shams El Arifeen, Michael J Dibley

**Affiliations:** ^1^ Sydney School of Public Health University of Sydney Sydney Australia; ^2^ Maternal and Child Health Division International Center for Diarrhoeal Diseases Research Dhaka Bangladesh

**Keywords:** unconditional cash transfer, mHealth, voice message, undernutrition

## Abstract

**Background:**

Inappropriate feeding practices, inadequate nutrition knowledge, and insufficient access to food are major risk factors for maternal and child undernutrition. There is evidence to suggest that the combination of cash transfer and nutrition education improves child growth. However, a cost-effective delivery platform is needed to achieve complete, population-wide coverage of these interventions.

**Objective:**

This study aimed to assess the feasibility, acceptability, and perceived appropriateness of an intervention package consisting of voice messaging, direct counseling, and unconditional cash transfers all on a mobile platform for changing perceptions on nutrition during pregnancy and the first year of a child’s life in a poor rural community in Bangladesh.

**Methods:**

We conducted a mixed-methods pilot study. We recruited 340 pregnant or recently delivered, lactating women from rural Bangladesh. The intervention consisted of an unconditional cash transfer combined with nutrition counseling, both delivered on a mobile platform. The participants received a mobile phone and BDT 787 per month (US $10). We used a voice messaging service to deliver nutrition-related messages. We provided additional nutrition counseling through a nutrition counselor from a call center. We carried out cross-sectional surveys at baseline and at the end of the study, focus group discussions, and in-depth interviews with participants and their family members.

**Results:**

Approximately 89% (245/275) of participants reported that they were able to operate the mobile phones without much trouble. Charging of the mobile handsets posed some challenges since only approximately 45% (124/275) households in our study had electricity at home. Approximately 26% (72/275) women reported they had charged their mobile phones at their neighbor’s house, while 34% (94/275) reported that they charged it at a marketplace. Less than 10% (22/275) of women reported difficulties understanding the voice messages or direct counseling through mobile phones, while only 3% (8/275) of women reported they had some problems withdrawing cash from the mobile bank agent. Approximately 87% (236/275) women reported spending the cash to purchase food for themselves and their children.

**Conclusions:**

The nature of our study precludes any conclusion about the effectiveness of the intervention package. However, the high coverage of our intervention and the positive feedback from the mothers were encouraging and support the feasibility, acceptability, and appropriateness of this program. Further research is needed to determine the efficacy and cost-effectiveness of mobile-based nutrition counseling and unconditional cash transfers in improving maternal and child nutrition in Bangladesh.

## Introduction

Maternal and child undernutrition remains one of the most serious health problems in low- and middle-income countries [1,2]. Maternal and child undernutrition together cause 35% of child deaths annually [2,3]and account for approximately 11% of disability-adjusted life years lost each year globally [3]. A global review of childhood undernutrition in 2012 revealed that Bangladesh was one of the top 20 countries, with a very high prevalence of stunted growth among children [4].

In recent years, Bangladesh has achieved a significant reduction in maternal and child undernutrition [5]. However, persistent socioeconomic inequality means that the overall prevalence of undernutrition has increased [6]. A recent paper reported that the prevalence of stunted growth is considerably higher in children from lower socioeconomic households than children from higher socioeconomic households [6]. Over the last two decades, the government has made substantial investments in the nutrition sector and implemented several large nutrition programs [7]. However, access and utilization of health services are still determined by income, education, and other social determinants of health, and frequently, the poor do not receive desired benefits from such programs [8,9].

Behavioral risk factors at the household level including, inappropriate maternal and infant feeding practices, insufficient access to food, and poor water and sanitation facilities lead to maternal and child undernutrition [3]. The current strategy of counseling for improved maternal diets and appropriate infant and young child feeding may not be enough to ensure reductions in maternal and child undernutrition without providing additional resources to the family, especially in poor and food-insecure households [10]. In Bangladesh and other low- and middle-income countries, nutrition education and counseling have been delivered in person by health workers and local volunteers through community-based platforms [1]. However, an insufficient number of health workers and low utilization of antenatal care by women from lower socioeconomic households challenges high-coverage delivery of nutrition education and counseling, especially among poor households [5,7].

Mobile phones are useful tools for sharing information. There is wide coverage of mobile phones in Bangladesh. In 2017, the number of active mobile phone subscribers in the country was 129.6 million [11]. With such high community penetration of mobile phones, messages through interactive voice response (IVR) systems or direct counseling via call centers could be a viable solution for the dissemination of nutritional behavior change messages in Bangladesh and other resource-poor settings. Direct counseling over mobile phones can also reach a larger population more cost-effectively than face-to-face counseling [12,13].

Cash transfers are increasingly being used, in both developmental and emergency contexts, to reduce poverty, to improve access to food and health and education outcomes. The provision of conditional cash transfers to poor households positively impacts overall health [14] and nutrition outcomes [15-17]. These programs can improve food consumption, food security, and dietary diversity, all of which are part of the causal pathway for child undernutrition. Compared to conditional cash transfers, there has been less research on unconditional cash transfers. However, a meta-analysis examining cash transfers on nutrition outcomes found small but nonsignificant impacts on height-for-age and also found that conditional programs had similar effects on unconditional cash transfers [18]. The arguments in favor of unconditional cash transfer programs are that the poor are rational actors and will invest some of their cash transfer on nutrition and health. Some also say that conditionality interferes with an individual’s right to choose [19]. Cash transfer programs are often associated with high administrative costs and require strong monitoring mechanisms to avert fraud. These pose significant barriers for implementing such interventions in low-resource settings.

We envisaged that combining unconditional cash transfers with mobile-based nutrition education and counseling would be a cost-effective intervention for reducing maternal and child undernutrition in poor households, with the potential for scale-up in Bangladesh and other resource-poor settings. This study sought to assess the feasibility, acceptability, and perceived appropriateness of an intervention package consisting of voice messaging, direct counseling, and unconditional cash transfers, all on a mobile platform, for changing perceptions on nutrition during pregnancy and the first year of a child’s life in a poor rural community in Bangladesh.

## Methods

### Study Design

We conducted a mixed-methods pilot study. The study received approval from the research review committee and the ethical review committee of the International Center for Diarrheal Disease Research, Bangladesh (icddr,b). We obtained written consent from all study participants before enrollment, household surveys, and qualitative interviews. We conducted the study in selected villages of two unions (local administrative unit) in Kendua Upazilla (subdistrict) under the Netrokona district. We purposively selected the villages and unions because of their overall low socioeconomic conditions. During the study period, more than 50% of the population was classified as poor and approximately 35% were classified as extremely poor [16]. According to the Bangladesh Bureau of Statistics definition, extremely poor households are those households whose total expenditures are less than or equal to the lower poverty line, which is commensurate with 1805 calories per person per day. The poor are those households whose total expenditures are less than or equal to the upper poverty line, which is commensurate with 2125 calories per person per day [20]. The study health workers made door-to-door visits across the study area covering 5429 households and identified pregnant women or recently delivered, lactating women. We recruited all women in our study area who were pregnant or had had a birth in the last 6 months and were currently lactating and expressed their willingness to participate in the assessment procedures. We followed up all participants for at least 6 months.

### Brief Description of the Intervention

#### Free Mobile Phone

Once registered, each woman received a mobile phone (Micromax X248 handset) with a standard connection from Banglalink, one of the largest mobile connection providers in the country. We also provided them with limited mobile credit of BDT 70 per month (US $0.89) for approximately 10 calls to the call center.

#### Prerecorded Mobile Voice Messages

We used life cycle stage-appropriate, prescheduled, voice messages from an IVR system called “Aponjon” for nutrition behavior change communication. Aponjon is a mobile-based mHealth service for expecting and new mothers in Bangladesh under the sponsorship of Mobile Alliance for Maternal Action. The Ministry of Health and Family Welfare has approved the content of Aponjon messages for wider distribution. A previous study, which assessed the efficacy of Aponjon messages, found no significant association between Aponjon messages and maternal healthcare behaviors. However, the study had inadequate power to detect any meaningful associations [21]. Aponjon covers topics like diet and care during pregnancy, breastfeeding, complementary feeding, care during child illnesses, and basic hygiene. We recorded all messages in Bangla, using a female voice. The duration of the messages was between 40 and 55 seconds. The Aponjon IVR system sent 2 voice messages per week to every participant for 24 weeks.

#### Direct Nutrition Counseling Through Mobile Phones

Trained nutrition counselors also provided nutrition support and information through a call center set up at the icddr,b. The counselors made biweekly calls to every participant for 24 weeks. The counselors listened to the women’s experience with their diet and feeding their children and offered suggestions and support. The call center provided nutrition-specific educational messages to all participants, depending on the women’s gestational age or age of the child. The counselors also regularly checked if the woman had been listening to and understood the voice messages sent to them through Aponjon, enquiring about the last message sent to the mother during their biweekly calls. Direct counseling focused on appropriate nutrition (amount of food, frequency of food) for pregnant and lactating women, exclusive breast feeding, complementary feeding, dietary diversity, appropriate care, health care seeking in pregnancy, hand washing, personal hygiene, sanitation, and other relevant topics.

#### Unconditional Cash Transfer Through Mobile Banking

We planned cash transfers of BDT 787 per month (US $10) and delivered them to the participants’ mobile phone. This amount represents approximately 17% of the average monthly income of the poorest 40% of rural households (approximately US $60 per month) [17]. Although we intended to make monthly transfers, we disbursed the total cash amount in 3 installments over 6 months due to logistical issues. Cash was transferred directly to the women’s bKash, mobile bank accounts, which greatly reduced the chance of illegal practices and also empowered the participating women. The study staff trained the participants about basic phone maintenance, receiving and listening to voice messages, and bKash mobile banking.

### Quantitative Data Collection

We used a single-arm experimental pre-post design. We conducted the baseline survey before we delivered the intervention. Then, after 8 months of implementing the intervention, a further end-line survey was conducted. We enrolled and interviewed 340 women in the pilot study during the baseline survey. However, we were able to interview only 275 (81%) women at the end of the project, mainly due to out-migration. We collected detailed information on household socioeconomic conditions, the use and usability of voice messaging and direct counseling, perceptions of the importance and usefulness of nutrition education, willingness to pay for such information services in future, understanding the content and knowledge retention, expenditure patterns of the project cash, compliance, barriers to mobile banking and nutrition education, and other related topics.

### Qualitative Data Collection

The qualitative study was conducted 8 months after the start of the intervention, between February and March 2016. Qualitative assessment included in-depth interviews, key informant interviews, and focus group discussions (FGD). For the qualitative interviews, we applied a purposive sampling strategy. Table 1 describes the methods and number of participants in the qualitative assessment. We developed separate semistructured guidelines for each type of respondent to guide the in-depth interviews and FGD. The guidelines were later translated into Bangla, in consultation with the local researchers, and were pretested with pregnant women, men, and a key informant. Based on the results of the pretest, the guidelines were adjusted.

### Data Analysis

Data captured during the household surveys were analyzed using Stata (StataCorp, 2015. Stata Statistical Software: Release 14. College Station, TX: StataCorp LP). We mostly used frequency analyses for all quantitative data. To measure the average time or willingness to pay, we calculated both the median and mean with SD. We used principal components analysis to construct a wealth index from available information on assets. We used information on ownership of household assets (such as TVs, radios, bicycles, motorcycles/scooters, tables, chairs, and wardrobes), housing materials (floor, wall, and roof materials), access to utilities (electricity, safe water, and clean energy), and house and land ownership to construct the household wealth index [22]. We later grouped the wealth index score into quintiles, with quintile 1 representing the poorest segment of the population and quintile 5, the wealthiest. To measure household food security, we used the Household Food Insecurity Access Scale, developed by the Food and Nutrition Technical Assistance Project [23].

**Table 1:** Methods, numbers, and types of respondents for the qualitative information.

Method and type of respondent		Number (N=21)
**In-depth interview**		
	Enrolled pregnant women	7
	Enrolled recently delivered mothers	7
	Husbands of enrolled women	5
**Focus group discussion**		
	Mothers-in-law of enrolled women	2

For qualitative data, all interviews were recorded using digital audio recorders. Audio recordings of all interviews and group discussions were transcribed verbatim in Bangla, the local language, by the research team. A qualitative expert (AA), who speaks Bangla, randomly checked the transcriptions against the audio recordings to ensure the quality of the interviews and transcription. We conducted thematic coding based on the data collection guidelines, but since our study was explorative, we were open to including any important themes that emerged in the transcript. We then discussed and summarized the text supporting each thematic code and have presented the findings using quotes and text tables.

## Results

### Quantitative Findings

#### Demographic Characteristics of the Women

We recruited 340 pregnant or recently delivered, lactating women between March 2015 and April 2015. Table 2 presents the baseline characteristics of all 340 participants who were recruited in the study, as well as of the 275 participants for whom we had complete information. Approximately half of the women were 15-24 years old. About one-third of the women had minimal education (ie, no education or incomplete primary level) but approximately two-thirds had completed the primary level of education. More than half of the women belonged to food-insecure households, with one-fifth belonging to severely food-insecure households. About 20% (65/340) of women were lost to follow-up in the end-line survey due to out-migration. However, we found no differences regarding sociodemographic characteristics between women who migrated out and women who remained in the study area.

#### Barriers to Using the Mobile Platform for Nutrition Counseling and Cash Transfer

Approximately 11% (30/275) of the women reported that they had faced some difficulty operating their mobile phones. Almost half the participants either had to go to their neighbor’s house or to a marketplace to charge their mobile phones. The median time duration when a mobile phone was switched off due to a dead battery was 2 days (Table 3).

#### Feasibility and Perceived Appropriateness of Nutrition Education Through Mobile Voice Messaging

About 2 out of 3 women reported missing at least one voice message from the Aponjon service over the 6-month study period. The mean and median number of missed voice messages was 3.1 (SD 2.4) and 2.0, respectively. Most women cited household responsibilities and dead batteries as the main reasons for failing to receive some of the voice message calls. Also, about a third of the participants reported having difficulty hearing the voice messages due to poor mobile network connections. Approximately 96% (263/275) of women reported that they did not know how to replay earlier messages they had received. Most women had no difficulties understanding the content of the messages. Nearly all of the mothers reported that they found the messages relevant to the stage of their pregnancy or the age of their child, and a similar percentage said the amount of information provided in each message was appropriate. They also found the frequency of messages (2 per week) adequate for their needs (Table 4).

#### Feasibility and Perceived Appropriateness of Direct Nutrition Counseling Through Mobile Phone

During the 6 month study period, the call center made a total of 4290 attempts (daily average of 32) and was successful in reaching the mothers in 81% (3475/4290) of cases. Each woman was scheduled to receive at least 12 calls from the call center, but approximately two-thirds of the women reported missing at least 1 call. The mean and median number of missed calls was 2.7 (SD 1.7) and 2 respectively. Being busy with household chores was cited as the most common reason for missing calls from the call center. About half of our participants reported making at least one call to the counselor, and the mean number of calls from the participants was 2.5 (SD 1.5). Approximately 95% (221/232) of our participants reported that they were satisfied with the counseling and the answers provided to their queries. However, unlike the voice message service, approximately 22% (60/275) of women said that biweekly calls were not sufficient. The women were asked to tell their preferred medium for nutrition communication. Approximately 62% (171/275) said they liked both communication channels, while one-third of women said they preferred direct counseling (Table 5).

#### Feasibility and Acceptability of Cash Transfers Through the Mobile Banking System

We also assessed the feasibility of transferring unconditional cash through the bKash mobile banking system. More than half of the participants opened their bKash mobile bank account in their name, while 35.6% (98/275) of the women had their account in their husband’s name (Table 6). The mean and median number of days to open a bkash account was 7.5 (SD 8.9) and 6 days, respectively.

**Table 2:** Characteristics of study participants at baseline and the end of the study.

Characteristic		Baseline (n=340), n (%)		End of study (n=275), n (%)	
**Age (years)**					
	15-24		179 (52.9)		140 (50.9)
	25-34		144 (42.4)		120 (43.6)
	35-44		16 (4.7)		14 (5.1)
**Women’s education**					
	No education		42 (12.4)		35 (12.7)
	Primary incomplete		84 (24.7)		69 (25.1)
	Primary complete		59 (17.4)		49 (17.8)
	Secondary incomplete		118 (34.7)		95 (34.5)
	Secondary or higher		36 (10.6)		26 (9.5)
**Husband’s education**					
	No education		107 (31.5)		90 (32.7)
	Primary incomplete		76 (22.4)		63 (22.9)
	Primary complete		43 (12.7)		35 (12.7)
	Secondary incomplete		72 (21.2)		54 (19.6)
	Secondary or higher		42 (12.4)		32 (11.6)
**Husband’s occupation**					
	Unskilled laborer		102 (30.0)		89 (32.4)
	Skilled worker		59 (17.4)		47 (17.1)
	Business/Trade		57 (16.8)		46 (16.7)
	Service holder		41 (12.1)		28 (10.2)
	Others		79 (23.2)		64 (23.3)
**Household wealth index**					
	Poorest		69 (20.5)		58 (21.1)
	Second		66 (19.6)		56 (20.4)
	Middle		68 (20.2)		56 (20.4)
	Fourth		67 (19.90)		51 (18.5)
	Richest		67 (19.9)		53 (19.3)
Household had at least one mobile		303 (89.1)		243 (88.4)	
Household had electricity		158 (46.5)		124 (45.1)	
**Household food security**					
	Secure		164 (48.7)		138 (50.2)
	Mildly food-insecure		9 (2.7)		5 (1.8)
	Moderately food-insecure		93 (27.6)		81 (29.5)
	Severely food-insecure		71 (21.1)		50 (18.2)

**Table 3:** Barriers in using mobile platform for nutrition counseling and cash transfer (n=275).

Barrier			n (%)
**Quality of the mobile set**			
	Very good		87 (31.6)
	Good		140 (50.9)
	Average		37 (13.4)
	Not good		11 (4.0)
Had difficulties receiving calls			16 (5.8)
Had difficulties making calls			30 (10.9)
**Place of mobile charging**			
		Home	142 (51.64)
		Neighbor’s house	72 (26.18)
		Marketplace	94 (34.18)

**Table 4:** Feasibility and perceived appropriateness of nutrition education through mobile voice messaging (n=275).

Voice message outcomes		n (%)
Received all voice messages^a^		101 (36.8)
Missed at least one voice message		173 (63.1)
Reasons for failing to receive voice messages^b^		
	Was busy with household chores	119 (66.5)
	Mobile was out of charge	60 (33.5)
Family members received messages (at least once)		87 (32.8)
**Had difficulties in hearing the messages (at least once)**		95 (34.7)
	Due to poor network	86 (90.5)
	Due to too much ambient noise	9 (9.5)
**Knew how to listen to an old message**		
	Yes	11 (4.0)
	No	263 (96.0)
Had difficulties understanding the messages (at least once)		22 (8.0)
Received text message mistakenly (at least once)		30 (10.9)
Messages were appropriate as per pregnancy and child age		265 (96.0)
The frequency of messages was appropriate		230 (78.8)
Amount of information in each message was appropriate		263 (96.0)

^a^The total number of voice messages was 48.

^b^Among women who reported missing at least 1 message.

**Table 5:** Feasibility and perceived appropriateness of direct nutrition counseling through mobile phone (n=275).

Call center outcomes			n (%)
Received all calls from the call center^a^			92 (33.4)
Ever missed any call from the call center			183 (66.5)
**Reasons for failing to receive a voice message**			
	Was busy with household chores	108 (59.0)	
	Mobile was out of charge	61 (33.3)	
Other family members spoke to the counselor (at least once)			80 (29.1)
Called the call center on her own (at least once)			151 (50.9)
**Reasons for not calling the call center**			
	Didn’t feel the need	114 (84.5)	
	Satisfied with the counseling and response to queries	221 (95.2)	
Counseling was appropriate as per pregnancy and child age			270 (98.2)
Frequency of counseling was appropriate			215 (78.2)
Direct counseling should be more frequent			60 (21.9)
Amount of information in each counseling was appropriate			270 (98.2)
**Preference for nutrition communication medium**			
	Voice messaging	14 (5.1)	
	Direct counseling from the call center	90 (32.7)	
	Both	171 (62.2)	

^a^The total number of counseling calls was 12.

In almost two-thirds of cases the women’s husbands were responsible for collecting the money. The mean and median times to go to the nearest bKash agent were approximately 24 (SD 14.7) and 20 minutes, respectively. Approximately 97% (264/272) of the participants reported having faced no problem withdrawing the money (Table 6). The mean and median times between the debit and withdrawal of cash were approximately 4 days (SD 6.8) and 2 days, respectively.

#### Willingness to Participate and Pay for Similar Services in the Future

We asked the participants whether or not they would be interested in receiving similar services in their next pregnancy and if they would be willing to pay for such services. Approximately 93% (255/275) and 92% (252/275) of the women expressed their willingness to receive voice message services and direct counseling in the future, respectively, and a similar percentage of women showed their willingness to pay and even join using their own mobile phones. On average they were willing to pay BDT 3 (US $0.04) for each voice message (median) and BDT 5 (US $0.06) for each counseling (median) call from the call center (Table 7).

### Qualitative Findings

Several significant themes emerged from our analyses, revealing the feasibility and acceptability of our intervention. We have illustrated the emergent themes with exemplary quotations. Many of the qualitative findings supported the quantitative findings presented above. Most participants referred to the voice messaging as a call from “Daktar Apa” (Doctor Sister) and the direct counseling from the call center as a call from “Pushti Apa” (Nutrition Sister).

#### Barriers to Operating Mobile Phones

Most participating women stated that they had no difficulties operating their mobile phones, even though some of them had no previous experience using mobile phones. Most women reported that on only a few occasions, they failed to receive the call, which supports our findings from the quantitative analysis. Most participants identified household responsibilities as a major factor related to missing calls. However, the majority successfully overcome this barrier by implementing their own, specific strategies, including organizing time and getting support from their families.

I used to keep myself free and wait on Monday and Wednesday around 8 in the morning for Daktar Apa’s call (voice message).

A pregnant woman

I told my mother about this program, and she would always make sure that I never miss a call.

A pregnant woman

#### Perceived Appropriateness of Voice Messaging and Direct Counseling

Women and family members felt the information provided was very important and beneficial for both mother and child. The majority perceived that they learned many new things. Some women believed they also experienced positive outcomes due to this intervention.

**Table 6:** Feasibility and acceptability of cash transfer through mobile banking from the end of study survey (n=275).

Cash transfer and mobile banking outcomes		n (%)
A mobile bank account in the participant’s name		150 (54.6)
A mobile bank account in the name of participant’s husband		98 (35.6)
A mobile bank account in the name of another family member		27 (9.8)
Difficulties understanding the cash transfer notification		73 (24.91)
**Reason for failing to understand the notification**		
	Failed to read or understand the short message service (SMS)	37 (50.68)
	Could not check the balance	34 (46.58)
**Person responsible for withdrawing the cash in the family**		
	Husband	169 (61.5)
	Self	70 (25.5)
	Other family members	36 (13.0)
**Main reasons for not going to withdraw cash by herself**		
	Restriction from the family	97 (48.0)
	Mobile agent office was too far	64 (31.7)
	Did not know where to withdraw cash	34 (16.8)
	No bank account on her name	19 (9.4)
	Difficulties withdrawing cash	8 (2.9)
**Person responsible for deciding how to spend the cash**		
	Herself	165 (60.6)
	Husband	56 (20.6)
	Both	40 (14.7)
**Expenditure pattern**		
	Purchase food for mother and child	236 (86.8)
	Purchased food for the family	42 (15.4)
	Used for another family purpose	14 (5.2)
	Used to buy livestock	11 (4.0)
	Savings	44 (16.2)

**Table 7:** Willing to participate and pay for similar services in future from the end of study survey (n=275).

Willingness to participate and pay	n (%)
Willing to receive nutrition education via voice message service in future	255 (93.1)
Willing to pay for voice messaging	234 (91.8)
Amount to pay for voice message, mean (SD); median	5.3 (6.5); 3.0
Willing to receive counseling via call center in future	252 (91.6)
Willing to pay for counseling	241 (95.6)
Amount to pay for each counseling, mean (SD); median	6.2 (6.1); 5.0
Willing to join similar services even if no phone is given	251 (91.2)
Will use my old phone	108 (43.0)
Would buy a new phone	64 (25.5)
Husband’s phone	71 (28.3)
Others	8 (3.2)

My babu (child) used to throw-up everything after feeding. Pushti Apa told me to tap on the back after each feeding. Now he does not throw up anymore.

A lactating woman

Now, I don’t have to go to Netrokona (district town) to seek consultations with doctors. Pushti Apa is fulfilling all her needs over the telephone.

Husband of a pregnant woman

I used to be skinny; now I have gained weight. I have eaten everything that they have advised me to eat.

A pregnant woman

The women understood the information provided through both voice messaging and direct counseling. Almost all the women reported that they did not have any problem understanding the language, the accent of the recorded voice, or the voice of the live counselor. The majority of women felt that both voice messaging and direct counseling were delivered in an easy to understand language.

I loved to listen to the messages, they spoke in such a nice language.

A pregnant woman

In response to the prompted question about the content of the last voice message or counseling from a nutrition counselor, the majority of women were able to recall the message’s content.

Daktar Apa told me (in the last call) that during diarrhoea we should not stop feeding our children and also continue the breastfeeding. They also told us to give oral saline and another medicine.

A lactating woman

Daktar Apa told me not to use any oil or water on newborn umbilicus. If the umbilicus (child’s) remain wet after 15 days, we should go and see a doctor.

A pregnant woman

Regarding appropriateness of message frequency, the majority felt 2 voice messages per week were sufficient, but the frequency of calls from the call center was inadequate.

A call after 15 or 20 days is sometime too late. There was an occasion when my child was vomiting, but I could not inform Apa (referring to the Counselor) before another two weeks.

A lactating woman

#### Preference Between Voice Messaging and Direct Counseling

Most women and family members liked the combination of voice messages and counseling by direct phone calls, and the opportunity to call the nutrition counselor when needed. They said each had a unique advantage. However, when asked to choose the best communications channel the majority chose direct counseling.

I liked the direct one (call), I could talk to Pushti Apa and ask for her advice if I have any problem with my child.

A lactating woman

I liked the direct call better; sometimes my wife doesn’t understand the voice message.

Husband of a lactating woman

But, there were a few participants who preferred the voice messages as they contained a wider range of topics and more detailed information.

The one that comes twice a week (voice message) is more useful. It provides information on different subjects.

A lactating woman

#### Barriers to Using Mobile Banking

Most women reported that the process of opening a bKash account was not complicated. However, the mandatory requirement of showing a national ID as a proof of identity did provide a barrier for some women.

My mother-in-law opened the bKash account for me, and she used my sister-in-law’s NID card as I had no NID card at that time.

A pregnant woman

In most cases, the participant’s husband went to the bKash agent to withdraw the mobile money. The majority said during pregnancy or immediately after childbirth, it is not customary for a woman to go outside, especially to the marketplace, which is far from the house. Also due to the women’s physical conditions, it might have been difficult to walk for long distances.

#### Use of Cash

Our findings revealed that for most families the cash received from the project was used for purchasing food, and on some occasions for treatments or medicines. The majority of women said they bought fruit, milk, eggs, vegetables, iron or calcium supplements, and suji (semolina) for themselves or their children, as advised by the nutrition counselor.

My husband bought me five egg-laying chickens. I can now eat eggs every day.

A pregnant woman

She used most of the money for herself like she bought banana, milk, and eggs for her.

Husband of a pregnant woman

A few families also used some of the money for other urgent needs, such as health care for their ill child and ill-mother. Some women invested some of the money in income-generating activities, such as buying poultry or saving money.

I have enough food in my house, so I spent the money for buying medicines and other household items.

A participating woman

I have saved around 1000 taka (US $12) and bought some poultries.

A participating woman

I consider the cash as a saving; if I can’t manage to get something for her from my own income, I will use the cash from the project.

Husband of a pregnant woman

#### Involvement of the Family

There was provision for providing a second number to the Aponjon voice message system, and many women provided their husband’s mobile number as the secondary number. However, very few of them reported receiving any voice messages on the secondary number. Some of the pregnant and lactating women tried to engage other family members in listening to the messages on their own.

My father-in-law, mother-in-law, sister-in-law – all appreciated this project. They are from old days and didn’t know a lot of things. They also have learnt many things from Daktar and Pushti Apa.

A lactating woman

I have learnt that my “abu” (grandchild) requires more nutritious food. It is important that we give him green leafy vegetables, chicken liver, khichuri (rice and lentil mixture). We didn’t know much about the importance of these foods.

Mother-in-law of a lactating woman

Our findings revealed widespread cooperation from the participating women’s husbands, who contributed to the decision-making regarding the spending of the money. No women reported any problems in using the money as they intended.

Since the family belongs to both husband and wife, it is wise to take decision together and spend accordingly.

A lactating woman

## Discussion

We found that our intervention of using mobile phones for nutrition counseling and cash transfer was a feasible and acceptable option for changing perceptions on nutrition during pregnancy and the first year of a child’s life among low-income families in rural Bangladesh. The women in the rural community found that this intervention was appropriate given their needs. Their willingness to participate and pay for the services further supports the acceptability of our intervention. The women were interested both in listening to the voice messages and in interacting with the nutrition counselor, which indicates that the combination of structured voice messaging and direct phone counseling could be the most effective communication strategy.

The current study also highlights the potential reach of mobile-based nutrition interventions in resource-constrained environments. Despite recruiting our study participants from a poor community and with one-third of our participants having no education or minimal education, we found that only a few of the study participants faced barriers to the use of mobile phones. Poor network or lack of mobile signal posed some difficulties in transmitting voice messages. However, the mobile networks in Bangladesh have grown significantly over recent years. Bangladesh currently has over 130 million mobile subscribers. Currently, mobile networks cover more than 99% of the country. Charging of the mobile phone also posed a barrier since almost half of the families did not have electricity at home. But most families were able to find a solution either by charging the phone at a neighbor’s house or taking the phone to the marketplace where there were commercial outlets for mobile charging.

We also found that cash transfer through the mobile banking system was a feasible means of distributing cash. In the majority of cases, women opened the account in their names. However, due to cultural and physical barriers (eg, distance) husbands were primarily responsible for withdrawing the money. The majority of the participants reported having faced no problem withdrawing the money. Regarding the use of cash, our study reported that one of the highest priorities for low-income families was purchasing food, although some families used the money for other urgent needs, such as healthcare for their ill child. Women from poor, but slightly better off, families tended to save some money to meet future needs or invest in income-generating activities, such as buying chickens.

Our results should be interpreted in the light of some of the limitations of our study. First, we used an existing voice messaging service, which was primarily developed for maternal health. However, we mitigated this by focusing on nutrition during the direct counseling. Second, we could not disburse the cash monthly as originally planned. There were some delays in the disbursement. Icddr,b is a research organization and not structured as a service-providing organization. There was no system in place to ensure regular monthly payments to a large number of participants outside the organization. This is unlikely to happen in program settings with organizations that have appropriate financial systems and experience to make regular cash transfer payments. The reduced frequency might have reduced the barriers; for example, it would have been a much higher opportunity cost for the families if they had to withdraw the money every month. Also, we do not know if the use of the cash would have been different if they received monthly payments, as originally planned. Third, the follow-up rate was not very high. We failed to follow up with 20% of the study participants at the end of the study. However, we did not find any differences between women who migrated from our study area and women who continued to reside there. Finally, in this study we did not aim to compare mobile counseling versus face-to-face counseling or to assess the impact of the of different communication strategies, and hence, there was no comparison group.

In Bangladesh, the government and different development organizations are showing strong interest in using nutrition counseling and cash transfer as tools to fight maternal and child undernutrition. Since we originally developed the concept of cash and counseling on a mobile platform, there is new evidence from Bangladesh supporting the combination of counseling and cash transfer as an intervention to reduce child undernutrition. A recently completed cluster-randomized controlled trial in rural Bangladesh, which followed the participants for 24 months, reported that the combination of cash transfers and nutrition behavior change communication decreased the rate of stunted growth by 7.3 percentage points, which is almost three times the average national decline [24]. The Government of Bangladesh, with assistance from World Bank, has recently launched a large program (Income Support Program for the Poorest, ISPP), which aims to reach 600,000 of the poorest households across Bangladesh with an objective to reduce poverty and improve child nutrition. The program will provide cash to the mothers, on the condition that they attend school and monthly nutrition sessions for mothers, including growth monitoring of the children [25]. However, a key limitation of these interventions is the intensity and level of human resources needed for face-to-face nutrition counseling, which makes it unlikely to be translated into large-scale programs. Also, most of the programs in Bangladesh, and elsewhere, especially in Latin America, use conditions for the cash transfer. The common conditions used in the programs include medical check-ups and school attendance [26-31]. However, health conditions only work best where the health system is ready to cater to the needs of an additional population who otherwise would not have come to the health center. With high community penetration of mobile phones, nutrition communication on mobile platforms is one of the most viable solutions for attaining high coverage for many low- and middle-income countries. Mobile-based communications can reach a larger population and help maintain the standard of counseling with simpler monitoring mechanisms [32]. Our pilot intervention of providing nutrition counseling through mobile platform thus provides an alternative approach that has the potential for upscale to reach across the country.

In Bangladesh, most cash transfer programs hand out cash directly to participants. In the ISPP program, the participating mothers will receive cash transfers into their post office accounts. We used mobile banking to disburse cash in our project. Mobile banking has been found to play a key role in financial inclusiveness for low-value transactions. Use of mobile cash transfers significantly reduces the costs to participants for obtaining the cash transfers and also reduces the implementing agency’s variable implementation costs [33]. In Bangladesh, mobile financial services are increasingly used across the country, and the daily transaction amount across all mobile banks was over BDT 47 million (US $603,000) in 2015 [34].

Although there is a great deal of interest and excitement surrounding nutrition counseling and cash transfer in Bangladesh, there is insufficient evidence documenting their effectiveness. Using the lessons learned in the pilot study for establishing a mobile-based system to send voice messages, provide direct counseling, and deliver cash transfer, we now plan to investigate the impact of this intervention on stunted growth in children in a larger randomized trial. We have already developed one such large study using a cluster-randomized controlled trial design where we aim to assess the effectiveness of mobile phones and nutrition behavior change communication, combined with unconditional cash transfers, for reducing the prevalence of stunted growth in children at 24 months. We will commence this 4-year study in 2018. The proposed trial will provide high-level evidence of the efficacy and cost-effectiveness of mobile phones and nutrition behavior change communication, combined with unconditional cash transfers, in reducing child undernutrition in a low-income and food-insecure population.

## Abbreviations

FGD: focus group discussions
IVR: interactive voice response
ISPP: Income Support Program for the Poorest

## References

[1] Bhutta ZA, Das JK, Rizvi A, Gaffey MF, Walker N, Horton S, Webb P, Lartey A, Black RE, Lancet Nutrition Interventions Review Group‚ the MaternalChild Nutrition Study Group (2013). Evidence-based interventions for improvement of maternal and child nutrition: what can be done and at what cost?. Lancet.

[2] Black RE, Allen LH, Bhutta ZA, Caulfield LE, de OM, Ezzati M, Mathers C, Rivera J, MaternalChild Undernutrition Study Group (2008). Maternal and child undernutrition: global and regional exposures and health consequences. Lancet.

[3] Black Robert E, Alderman Harold, Bhutta Zulfiqar A, Gillespie Stuart, Haddad Lawrence, Horton Susan, Lartey Anna, Mannar Venkatesh, Ruel Marie, Victora Cesar G, Walker Susan P, Webb Patrick, Maternal and Child Nutrition Study Group (2013). Maternal and child nutrition: building momentum for impact. Lancet.

[4] Stevens G, Finucane Mariel M, Paciorek Christopher J, Flaxman Seth R, White Richard A, Donner Abigail J, Ezzati Majid, Nutrition Impact Model Study Group (Child Growth) (2012). Trends in mild, moderate, and severe stunting and underweight, and progress towards MDG 1 in 141 developing countries: a systematic analysis of population representative data. Lancet.

[5] (2014). The DHS Program.

[6] Huda T, Hayes Alison, El Arifeen Shams, Dibley Michael J (2018). Social determinants of inequalities in child undernutrition in Bangladesh: A decomposition analysis. Matern Child Nutr.

[7] (2015). World Bank Study.

[8] Celik Y, Hotchkiss DR (2000). The socio-economic determinants of maternal health care utilization in Turkey. Soc Sci Med.

[9] Gwatkin DR, Bhuiya A, Victora CG (2004). Making health systems more equitable. Lancet.

[10] Ruel M, Alderman Harold, Maternal and Child Nutrition Study Group (2013). Nutrition-sensitive interventions and programmes: how can they help to accelerate progress in improving maternal and child nutrition?. Lancet.

[11] Azam Sifat-E, Mustafa K. Mujeri (2018). Interoperability of Digital Finance in Bangladesh: Challenges and Taking-Off Options. http://inm.org.bd/wp-content/uploads/2018/06/Working-Paper-54.pdf.

[12] Liao Y, Wu Q, Tang J, Zhang F, Wang X, Qi C, He H, Long J, Kelly BC, Cohen J (2016). The efficacy of mobile phone-based text message interventions ('Happy Quit') for smoking cessation in China. BMC Public Health.

[13] Zhao J, Freeman B, Li M (2016). Can Mobile Phone Apps Influence People's Health Behavior Change? An Evidence Review. J Med Internet Res.

[14] Rasella D, Aquino Rosana, Santos Carlos A T, Paes-Sousa Rômulo, Barreto Mauricio L (2013). Effect of a conditional cash transfer programme on childhood mortality: a nationwide analysis of Brazilian municipalities. Lancet.

[15] Fernald L, Gertler Paul J, Neufeld Lynnette M (2009). 10-year effect of Oportunidades, Mexico's conditional cash transfer programme, on child growth, cognition, language, and behaviour: a longitudinal follow-up study. Lancet.

[16] Leroy JL, García-Guerra A, García R, Dominguez C, Rivera J, Neufeld LM (2008). The Oportunidades program increases the linear growth of children enrolled at young ages in urban Mexico. J Nutr.

[17] Fernald L, Gertler Paul J, Neufeld Lynnette M (2008). Role of cash in conditional cash transfer programmes for child health, growth, and development: an analysis of Mexico's Oportunidades. Lancet.

[18] Manley J (2013). , S. Gitter, and V. Slavchevska, How Effective are Cash Transfers at Improving Nutritional Status?. World Development.

[19] Robertson L, Mushati Phyllis, Eaton Jeffrey W, Dumba Lovemore, Mavise Gideon, Makoni Jeremiah, Schumacher Christina, Crea Tom, Monasch Roeland, Sherr Lorraine, Garnett Geoffrey P, Nyamukapa Constance, Gregson Simon (2013). Effects of unconditional and conditional cash transfers on child health and development in Zimbabwe: a cluster-randomised trial. Lancet.

[20] Bangladesh Bureau of Statistics, Dhaka, Bangladesh.

[21] Alam M, D'Este Catherine, Banwell Cathy, Lokuge Kamalini (2017). The impact of mobile phone based messages on maternal and child healthcare behaviour: a retrospective cross-sectional survey in Bangladesh. BMC Health Serv Res.

[22] Vyas S, Kumaranayake L (2006). Constructing socio-economic status indices: how to use principal components analysis. Health Policy Plan.

[23] Food and Agriculture Organization of the United Nations.

[24] Ahmed, Akhter U., Hoddinott John F., Roy Shalini, Sraboni Esha, Quabili Wahidur R, Margolies Amy (2016). Which Kinds of Social Safety Net Transfers Work Best for the Ultra Poor in Bangladesh?: Operation and Impacts of the Transfer Modality Research Initiative. https://catalog.princeton.edu/catalog/10397293.

[25] Ferre Celine, Sharif Iffath (2014). Can Conditional Cash Transfers Improve Education and Nutrition Outcomes for Poor Children in Bangladesh? Evidence from a Pilot Project. Policy Research Working Paper;No. 7077.

[26] Fernald L, Gertler Paul J, Neufeld Lynnette M (2008). Role of cash in conditional cash transfer programmes for child health, growth, and development: an analysis of Mexico's Oportunidades. Lancet.

[27] Leroy JL, García-Guerra A, García R, Dominguez C, Rivera J, Neufeld LM (2008). The Oportunidades program increases the linear growth of children enrolled at young ages in urban Mexico. J Nutr.

[28] Fernald LCH, Gertler PJ, Neufeld LM (2009). 10-year effect of Oportunidades, Mexico's conditional cash transfer programme, on child growth, cognition, language, and behaviour: a longitudinal follow-up study. Lancet.

[29] Paxson C, Schady Norbert (2010). Does money matter? The effects of cash transfers on child development in rural Ecuador. Econ Dev Cult Change.

[30] Segura-Pérez S, Grajeda R, Pérez-Escamilla R (2016). Conditional cash transfer programs and the health and nutrition of Latin American children. Rev Panam Salud Publica.

[31] Baird S (2011). , C. McIntosh, and B. Özler, Cash or condition? Evidence from a cash transfer experiment. The Quarterly Journal of Economics.

[32] Lester R, Mills Edward J, Kariri Antony, Ritvo Paul, Chung Michael, Jack William, Habyarimana James, Karanja Sarah, Barasa Samson, Nguti Rosemary, Estambale Benson, Ngugi Elizabeth, Ball T Blake, Thabane Lehana, Kimani Joshua, Gelmon Lawrence, Ackers Marta, Plummer Francis A (2009). The HAART cell phone adherence trial (WelTel Kenya1): a randomized controlled trial protocol. Trials.

[33] Aker J, Boumnijel R, McClelland A, Tierney N (2016). Payment Mechanisms and Antipoverty Programs: Evidence from a Mobile Money Cash Transfer Experiment in Niger. Economic Development and Cultural Change.

[34] Parvez Jaheed, Islam Ariful, Woodard Josh (2015). Mobile Financial Services in bangladesh A Survey of Current Services, Regulations, and Usage in Select USAID Projects.

